# Cooler Temperatures Destabilize RNA Interference and Increase Susceptibility of Disease Vector Mosquitoes to Viral Infection

**DOI:** 10.1371/journal.pntd.0002239

**Published:** 2013-05-30

**Authors:** Zach N. Adelman, Michelle A. E. Anderson, Michael R. Wiley, Marta G. Murreddu, Glady Hazitha Samuel, Elaine M. Morazzani, Kevin M. Myles

**Affiliations:** Fralin Life Science Institute and Department of Entomology, Virginia Tech, Blacksburg, Virginia, United States of America; United States Army Medical Research Institute of Infectious Diseases, United States of America

## Abstract

**Background:**

The impact of global climate change on the transmission dynamics of infectious diseases is the subject of extensive debate. The transmission of mosquito-borne viral diseases is particularly complex, with climatic variables directly affecting many parameters associated with the prevalence of disease vectors. While evidence shows that warmer temperatures often decrease the extrinsic incubation period of an arthropod-borne virus (arbovirus), exposure to cooler temperatures often predisposes disease vector mosquitoes to higher infection rates. RNA interference (RNAi) pathways are essential to antiviral immunity in the mosquito; however, few experiments have explored the effects of temperature on the RNAi machinery.

**Methodology/Principal Findings:**

We utilized transgenic “sensor” strains of *Aedes aegypti* to examine the role of temperature on RNA silencing. These “sensor” strains express EGFP only when RNAi is inhibited; for example, after knockdown of the effector proteins Dicer-2 (DCR-2) or Argonaute-2 (AGO-2). We observed an increase in EGFP expression in transgenic sensor mosquitoes reared at 18°C as compared with 28°C. Changes in expression were dependent on the presence of an inverted repeat with homology to a portion of the EGFP sequence, as transgenic strains lacking this sequence, the double stranded RNA (dsRNA) trigger for RNAi, showed no change in EGFP expression when reared at 18°C. Sequencing small RNAs in sensor mosquitoes reared at low temperature revealed normal processing of dsRNA substrates, suggesting the observed deficiency in RNAi occurs downstream of DCR-2. Rearing at cooler temperatures also predisposed mosquitoes to higher levels of infection with both chikungunya and yellow fever viruses.

**Conclusions/Significance:**

This data suggest that microclimates, such as those present in mosquito breeding sites, as well as more general climactic variables may influence the dynamics of mosquito-borne viral diseases by affecting the antiviral immunity of disease vectors.

## Author Summary

Although a link between the increased susceptibility of mosquitoes for arthropod-borne viruses and exposure to lower rearing temperatures has been known for many years, the molecular basis of this has remained unknown. We investigated this phenomenon using an engineered strain of mosquito where the expression of a reporter was dependant on the status of the RNA interference pathway (RNAi). Our studies indicate a correlation between the virus-susceptibility phenotype and temperature-dependent deficiencies in antiviral immunity. Specifically, we demonstrate that RNAi, a critical antiviral immune pathway in mosquito vectors of human disease, is impaired in insects reared at cooler temperatures. This suggests for the first time a molecular explanation for previously described observations, findings that may lead to a better understanding of how global climate change will affect the transmission of mosquito-borne viruses, and new criteria for evaluating genetic control strategies based on RNAi. Our studies also suggest a novel mechanism for arbovirus adaptation to otherwise incompetent vector species.

## Introduction

There is a great deal of uncertainty concerning how climate change will affect the distribution and incidence of infectious diseases, particularly vector-borne diseases. Although there have been predictions of increased vector-borne disease with increasing global temperatures [1], modeling such changes is difficult because of the complex relationships between vector-borne diseases and temperature [2], [3], [4], [5], [6]. For example, higher temperatures can shorten the time it takes mosquitoes to become competent vectors of human disease agents [7], [8], but also shorten the mosquito lifespan; thus, decreasing their ability to serve as successful vectors [5].

Temperature-dependent effects on the susceptibility of mosquito vectors to viral infection also contribute to the complexity of disease transmission. An inverse relationship appears to exist between the temperature at which mosquito larvae undergo development and their future susceptibility to virus infection [9], [10], [11]. Lower temperatures in particular have been shown to adversely affect a vector's ability to modulate viral infection [12], [13], [14] and increase rates of transovarial transmission [15]. Kramer et al [12] demonstrated that infection levels of western equine encephalomyelitis virus (WEEV; genus *Alphavirus*) in *Culex (Cx.) tarsalis* decreased as a function of increasing temperature. These researchers hypothesized that at higher temperatures, mosquitoes were better able to modulate virus infection. This ability to modulate virus infection was heritable, suggesting a genetic basis for the phenotype. Similar findings were made by Kay and Jennings [14] using Ross River virus (RRV; genus *Alphavirus*) in *Ochelerotatus vigilax* reared at 18°C. Turell [10] also found significantly higher rates of disseminated infections for both Rift Valley fever virus (RVFV; genus *Phlebovirus*) and Venezuelan equine encephalomyelitis virus (VEEV, genus *Alphavirus*) following the ingestion of an infectious blood meal when mosquitoes were reared at 19°C as compared to 26C. Most recently, Westbrook et al. [11] found that the infectivity of CHIKV for *Ae. albopictus* increased with decreasing rearing temperature (18°C>24°C>32°C). Despite these observations, no mechanism has been implicated that might explain how lower temperatures predispose mosquito vectors to higher infection rates.

It is clear that small RNA pathways are critical in controlling virus infections of the mosquito host [16], [17], [18], [19]. Infection of the mosquito with an arbovirus results in the production of two different classes of small RNAs; 21 nt small interfering RNAs (vsiRNAs) [16] and 24–30 nt piwi-interacting RNAs (vpiRNAs), with the latter possessing a clear ping-pong signature [19], [20]. Suppressing production of viral small RNAs in the mosquito leads to increased viral replication and mortality [16]. The generation of vsiRNAs depends on the activity of dicer-2 (DCR-2) [21]. However, processing by DCR-2 alone is insufficient to control virus replication. Rather, this depends on the slicer activity of Argonaute 2 (AGO-2), a component of the RNA induced silencing complex (RISC) [22], [23], [24]. The RISC undergoes a maturation process in which one strand of a siRNA duplex is selected as a guide, directing the slicer activity of AGO-2 to RNA targets present within the cell. The specific molecules involved in the biogenesis and activity of vpiRNAs have not yet been identified.

Here, we correlate loss of RNA silencing in mosquitoes reared at 18°C and increased susceptibility of *Aedes* spp. mosquitoes for infection with chikungunya virus (CHIKV; genus *Alphavirus*) and yellow fever virus (YFV; genus *Flavivirus*), suggesting a molecular basis for previous observations of increased transmission of viral pathogens by disease vector mosquitoes exposed to cooler temperatures.

## Materials and Methods

### Mosquitoes and viruses

All mosquito strains [*Ae. aegypti* Liverpool, *kh^w^* (white eye) and all transgenic strains, *Ae. albopictus* Wise County strain] were reared in environmental chambers with a light cycle of 14∶10 (day∶night) and humidity of 80%. For all experiments, mosquitoes were hatched under vacuum for ∼30 minutes, counted, and dispensed (n = ∼400) into 4 L of water pre-chilled at 18°C or pre-warmed at 28°C. Unless otherwise stated in the text, all stages of development occurred at the indicated temperature. CHIKV strain 37797 was produced from an infectious clone as described previously [19]. YFV (Asibi strain) was obtained from the Centers for Disease Control and Prevention. Recombinant Sindbis (SIN) viruses containing *Ae. aegypti dcr-2* or *ago-2* fragments were as described in [25].

### Per os infection and infectivity assays

Assays were performed as described previously [26], with the following exception. As vertebrate cells inoculated with small quantities of virus require additional infectious cycles before observation of cytopathic effects (CPE), rapidly proliferating vertebrate cell monolayers may begin to deteriorate after too many doublings and the exhaustion of medium. Deterioration of cell monolayers inoculated with relatively small quantities of virus may obscure scoring of CPE. To militate against this, clarified mosquito homogenates were inoculated onto C6/36 mosquito cell monolayers and placed at 28°C for 48 hours; the supernatant from inoculated C6/36 cells was added to BHK-21 cell monolayers, which were then scored for cytopathic effects.

### Generation of transgenic mosquitoes

Transgenic *Ae. aegypti* were generated as described previously [25], [27]. Briefly, freshly deposited *Ae. aegypti* (*kh^w^* strain) embryos were microinjected with 500 µg/µl Donor plasmid and 300 µg/µl pKhsp82 *Mos*1 Helper plasmid. The 3×P3-RG Donor plasmid was identical to that described in [25], with the exception that the final 3×P3-EGFP inverted repeat cassette was deleted. Surviving individuals were crossed with the parental strain and progeny screened for the presence of DsRED fluorescence in the eyes.

### Real time quantitative PCR

SYBR Green-based qPCRs for EGFP, *dcr-2* and *ago-2* mRNA levels were performed as described in [25]. Detection and quantitation of CHIKV (+) strand RNA was performed as described in [19], [28]. YFV RNA was quantified with a standard TaqMan® assay as recommended by the manufacturer (Life Technologies, Grand Island, NY).

### Small RNA library preparation, sequencing and analysis

Small RNA libraries derived from head tissue of transgenic sensor strain mosquitoes were constructed using Illumina's small RNA sample prep kit and sequenced on an Illumina GAII (single replicate per temperature). Small RNA libraries derived from whole female *Ae. aegypti* infected with CHIKV (three biological replicates per temperature) were barcoded and constructed with Illumina's TruSeq™ small RNA prep kit and sequenced on an Illumina HiSeq. All small RNA reads were mapped to the sensor transgene or the CHIKV genome using Bowtie (v12.7) [29] after removal of the 3′ adapter sequence. Differential expression of viRNAs were calculated as described in Morazzani et al [19]. All small RNA libraries described in this study are available for download from the Gene Expression Omnibus (GEO accession # GSE46204).

## Results

### Cool temperature inhibits RNA silencing

**Figure 1:**
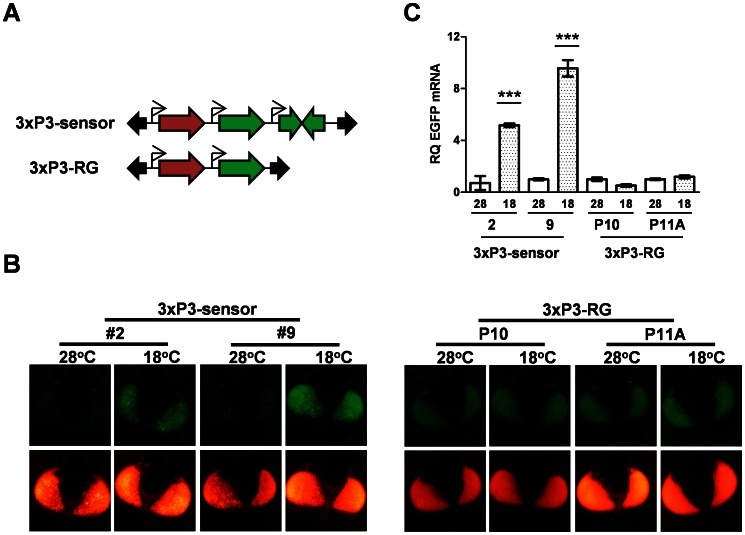
Rearing at 18°C activates transgenic RNAi sensor strain mosquitoes. (**A**) Schematic representation of the two transgenic constructs. 3×P3-DsRED (red block arrows), 3×P3-EGFP/3×P3-EGFPir (green block arrows), and *Mos1* right and left arms (black block arrows) are indicated. (**B**) Photographs of typical individuals from *Ae. aegypti* 3×P3-sensor or 3×P3-RG transgenic strains following rearing at 18°C or 28°C using a Leica EGFP (top panel) or DsRED filter (bottom panel). (**C**) Real-time qPCR displaying changes in EGFP mRNA levels within each of four transgenic strains (two sensor strains, two 3×P3-RG strains) following rearing at 18°C (18) or 28°C (28). Error bars indicate the standard deviation among three biological replicates; *** indicates significance at the p<0.001 level as determined by two-tailed Student's t-test.

**Figure 2:**
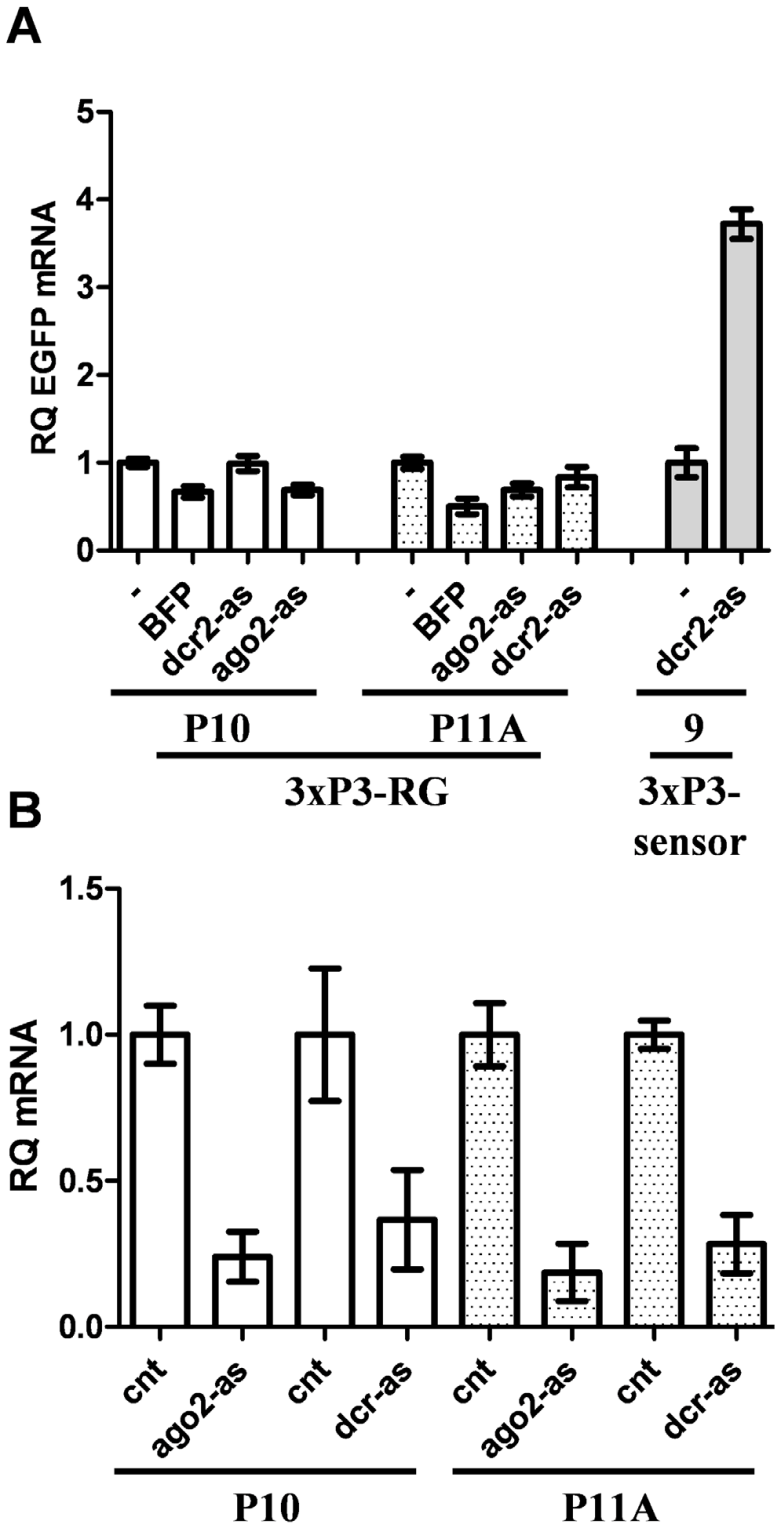
Loss of DCR-2 or AGO-2 transcripts does not affect EGFP expression in transgenic 3×P3-RG lines. (**A**) Real-time qPCR displaying changes in EGFP mRNA levels following knock-down of *ago-2* or *dcr-2* transcripts in two 3×P3-RG transgenic lines and one 3×P3-sensor line. (**B**) Real-time PCR results confirming successful knockdown of *ago*-2 and *dcr*-2 transcripts. Error bars indicate one standard deviation corresponding to technical variation for a representative biological replicate.

We previously described two different *Ae. aegypti* transgenic “sensor” strains that express both EGFP and an inverted repeat derived from EGFP in an eye-specific manner; with knockdown of DCR-2 or AGO-2 resulting in increased EGFP expression (Fig. 1A and [25]). In order to determine if rearing mosquitoes at a cooler temperature adversely affects the ability of the RNAi machinery to silence the EGFP transgene, we reared the two independent sensor strains at 18°C or 28°C. We observed a loss of silencing of the EGFP transgene in both strains when mosquitoes were reared at 18°C (Fig. 1B). To verify that the observed increase in EGFP fluorescence was due to a corresponding increase in the steady-state levels of EGFP mRNA, we performed real-time qPCR on cDNA obtained from the heads of mosquitoes reared at 18°C or 28°C. In both sensor strains reared at the lower temperature, we observed a 4–8 fold increase in EGFP mRNA levels (Fig. 1C). To verify that this was due to a temperature-dependent effect on RNAi, and not non-specific transcriptional or post-transcriptional changes affecting the expression of the transgenes, we generated additional transgenic strains (termed 3×P3-RG) carrying a similar construct as our sensor but without the inverted repeat sequence (Fig. 1A). Two different lines were established, P10 and P11A. As expected, neither line P10 nor P11A exhibited any changes in EGFP fluorescence (Fig. 1B) or EGFP mRNA levels (Fig. 1C) when reared at 18°C. Unlike 3×P3-sensor mosquitoes, the 3×P3-RG transgenic lines did not show any change in EGFP protein or mRNA levels with the loss of DCR-2 or AGO-2 (Fig. 2A, 2B), suggesting that the inverted repeat sequence targeting EGFP is essential for both RNAi-based silencing of EGFP and temperature-dependent loss of silencing.

**Figure 3:**
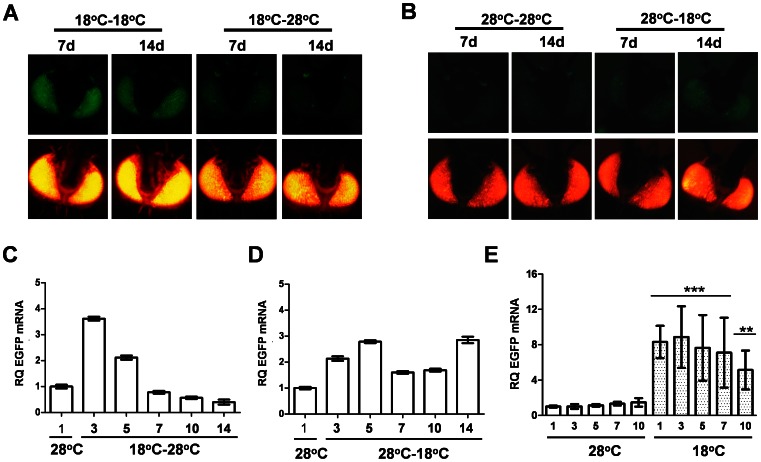
Low-temperature activation of RNAi sensor mosquitoes is reversible and can be induced in adults. Photographs taken at 7 or 14 days post emergence (7 d or 14 d) of typical individuals from *Ae. aegypti* RNAi sensor strain #2 following rearing at 18°C (**A**) or 28°C (**B**). Adult females were transferred to the indicated temperature at 1 day post-emergence; photographs are EGFP (top panel) or DsRED (bottom panel). Real-time qPCR of EGFP mRNA levels in 3×P3-sensor mosquito heads following rearing at 18°C (**C**) or 28°C (**D**), with newly emerged adults held at the alternate temperature for the indicated number of days. Error bars indicate one standard deviation corresponding to technical variation for a representative biological replicate. (**E**) Real-time qPCR of EGFP mRNA levels in transgenic RNAi sensor heads following rearing at 18°C or 28°C, with mosquitoes remaining at the same temperature as adults. Error bars indicate the standard deviation among three biological replicates; *** indicates significance at the p<0.001 level as determined by two-tailed Student's t-test.

In order to determine if temperature-dependent loss of silencing could be rescued, mosquitoes reared at 18°C were transferred to 28°C one-day post emergence. EGFP mRNA levels were silenced by seven days (Fig. 3A, 3C), whereas mosquitoes that remained at 18°C failed to silence EGFP (Fig. 3E). This indicated that the RNAi pathway is not irrevocably damaged during development at cooler temperatures. Likewise, rearing mosquitoes at 28°C, with a subsequent shift of the newly emerged adults to 18°C resulted in a partial loss of silencing (Fig. 3B, 3D), suggesting that this phenomenon is not necessarily fixed to a specific developmental stage.

**Figure 4:**
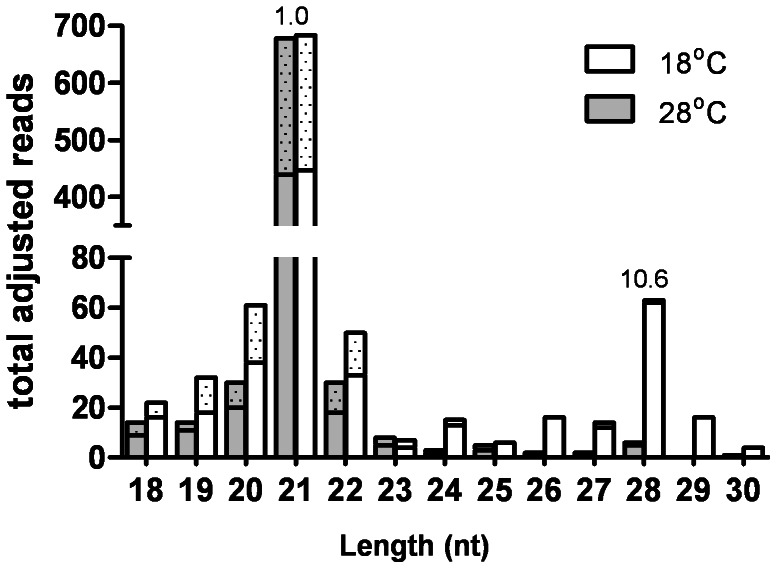
Small RNA sequencing from 3×P3-sensor heads. Histogram displaying the length distribution of EGFP-derived small RNAs from 3×P3-sensor mosquito heads following rearing at 18°C or 28°C. Numbers above bars indicates fold increase from 28°C to 18°C. Sense (solid bar) and antisense (cross-hatched) siRNA totals are displayed in the same stack for each sample.

In order to determine if rearing at cooler temperatures perturbed the biogenesis of small RNAs, we sequenced small RNAs isolated from the heads of sensor strain mosquitoes reared at 28°C or 18°C (Fig. 4). Small RNA biogenesis was not affected by the lower temperature, suggesting that the observed effects on RNA silencing occur downstream of the initial dicing step. Interestingly, we observed an increase in 28 nt small RNAs derived from the EGFP inverted repeat transgene in the 18°C cohort, suggesting the possibility of increased targeting by the piRNA pathway (Fig. 4).

To determine if the observed loss of transgene silencing correlated with increased susceptibility to viral infection, we fed sensor strain mosquitoes reared at 28°C or 18°C blood meals containing equivalent amounts of CHIKV. When infectivity was assayed eight days after the infectious blood meal, sensor mosquitoes reared at 18°C had significantly (Fisher's exact test, P<0.03) higher infection rates with CHIKV in comparison with the cohorts reared at 28°C (Fig. S1).

### Cool temperature increases the susceptibility of Ae. aegypti to CHIKV infection

**Figure 5:**
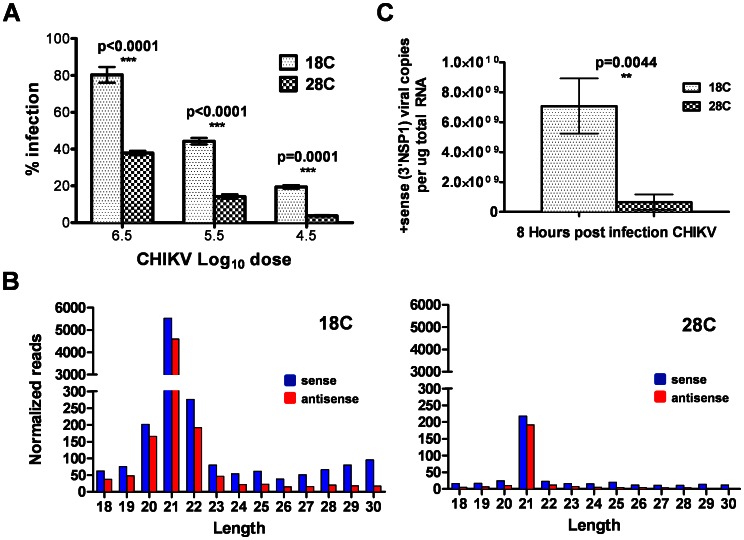
CHIKV infection, RNA production and small RNA biogenesis following rearing at 18°C or 28°C. (**A**) Infectivity of CHIKV for *Ae. aegypti* Liverpool strain following *per os* challenge. Each bar represents the average of three biological replicates of 27–50 mosquitoes each. Small RNA size distributions of CHIKV vsiRNAs and vpiRNAs (**B**) and absolute quantitation of CHIKV (+) strand RNA (**C**) at 8 hrs post injection into *Ae. aegypti* Liverpool strain females reared at 18°C or 28°C. Error bars indicate the standard deviation amongst three biological replicates. Significance was assessed via a two-tailed Student's t-test.

To determine if cooler temperatures also increase susceptibility of non-transgenic mosquitoes to CHIKV, Liverpool strain *Ae. aegypti* were offered blood meals containing serial dilutions of the virus. Eight days after challenge, the number of CHIKV-infected mosquitoes was determined. Even though both groups were held at 28°C for the duration of the viral extrinsic incubation period, prior exposure to a temperature of 18°C substantially increased the number of CHIKV-infected mosquitoes following ingestion of the blood meal, as compared with those reared at 28°C (Fig. 5A). Next, we injected mosquitoes reared at 18°C or 28°C with equivalent amounts of virus, transferred both groups to 28°C, and processed 8 hours later; this time point was chosen specifically to limit the number of infectious cycles. This route of infection also bypassed the midgut, to verify that the temperature dependent effect was systemic, and not localized to the midgut tissue. Sequencing small RNA populations from these mosquitoes revealed significantly increased production of vsiRNAs (exact Poisson test, *P*<0.0001) and vpiRNAs (exact Poisson test, *P*<0.0001) in cohorts reared at the colder temperature (Fig. 5B). Real-time quantitative PCR analysis performed on mosquitoes reared at 18°C indicated significantly higher levels of viral mRNA than in those reared at 28°C (Fig. 5C). Thus, despite their increased presence in mosquitoes reared at 18°C, the dicer products in these mosquitoes were not effective at controlling virus replication, likely due to impairment of RNAi at a downstream step [21], [22], [30].

**Figure 6:**
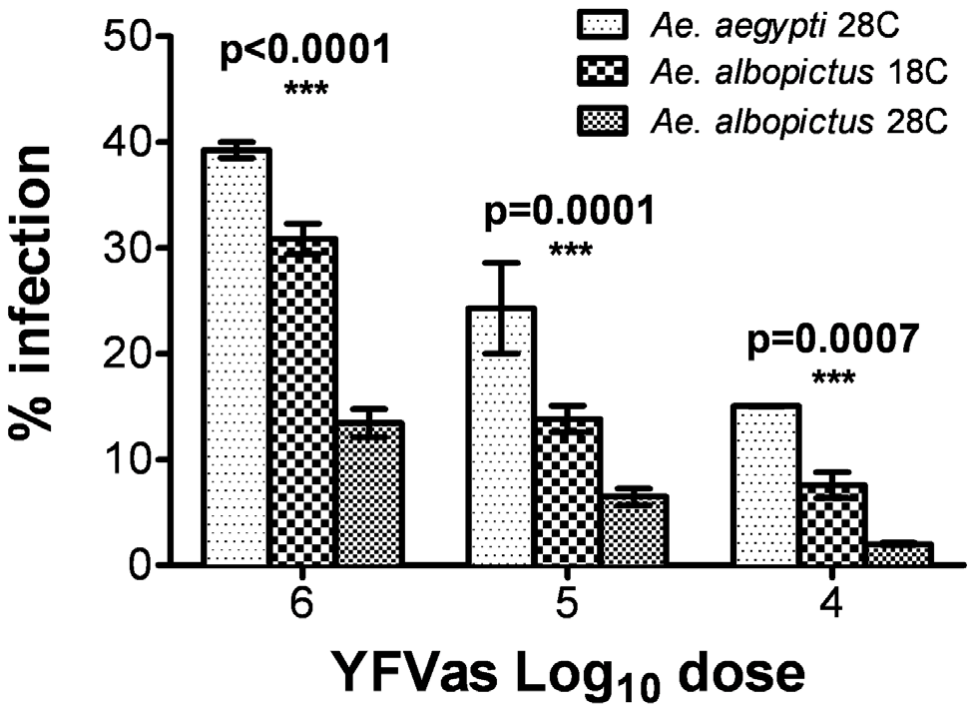
YFV infection of mosquitoes following rearing at 18°C or 28°C. Infectivity of YFV for *Ae. aegypti* (Lvp strain) and *Ae. albopictus* (Wise County) reared at 18°C or 28°C. Each bar represents the average of three biological replicates of 39–50 mosquitoes each; error bars indicate one standard deviation. Significance was assessed via a two-tailed Student's t-test.

To confirm that these findings were not virus or host-specific, we offered infectious bloodmeals containing YFV to a recently colonized *Ae. albopictus* strain (2008) reared at 18°C or 28°C, and compared these results to those obtained in *Ae. aegypti* reared at 28°C. Similar to our previous results, prior exposure to a temperature of 18°C significantly increased YFV infections in *Ae. albopictus* ingesting blood meals containing the virus (Fig. 6). Our results also indicated that the Liverpool strain of *Ae. aegypti* was more susceptible to infection with YFV when reared at 28°C than was the *Ae. albopictus* strain reared at 28°C or 18°C. However, the difference was not as great between *Ae. aegypti* reared at 28°C and *Ae. albopictus* reared at 18°C (Fig. 6).

## Discussion

Our data suggest post-dicer inhibition of RNA silencing in disease vector mosquitoes subjected to low temperature (18°C). Mosquitoes reared at the same low temperature and subsequently infected with CHIKV or YFV, proved significantly more susceptible to these viruses than mosquitoes reared at 28°C. These results also corresponded with significantly increased virus replication in mosquitoes exposed to the lower temperature. In mosquitoes infected with CHIKV, increased virus replication occurred despite a significant rise in production of vsiRNAs, suggesting a diminished potency for these dicer products after mosquitoes were reared at the colder temperature. These results are consistent with those of others that have shown the production of vsiRNAs is insufficient to control virus replication in the absence of AGO-2 mediated slicing [21], [22], [30]. Thus, we propose that the temperature-dependent effects observed in both transgenic and non-transgenic mosquitoes are due to either direct or indirect inhibition of AGO-2-dependent slicing. Interestingly, temperature-dependent defects in RNAi have also been described in several plant species [31], [32]. However, in contrast with our results these studies found that dicing of viral dsRNAs was inhibited in plants exposed to cooler temperatures. Nevertheless, the similarity of the plant phenotypes to those described here suggests a convergent evolutionary response to lower than optimal temperatures in both plants and insects.

Previous work by our group has demonstrated that suppression of the RNAi response by the protein B2 increases the midgut infection rate for arboviruses [16]. Similar results were observed by others following silencing of DCR-2 or AGO-2 [17], [18]. These data emphasize the idea that arbovirus entry and uncoating into midgut cells is followed by an effective RNAi response that eliminates many or all of these initial events so that a productive midgut infection is avoided. In our experiments, mosquitoes fed an infectious bloodmeal were treated in an identical manner *after* virus exposure. Thus, we expect the effects of temperature were limited to the time of virus exposure only. A reduction in RNA silencing at this time would be expected to reduce the ability of initially infected cells to mount a response early in the infectious process; the end result being an increase in the number of productive midgut infections. Once the arbovirus has established this initial foothold, the RNAi response, even if functioning normally might not be able to completely clear the infection, thus, these mosquitoes remain infected for life.

Our data suggest that exposure to cooler temperatures after virus infection would also reduce RNAi activity; however, such temperatures would also reduce the replicative capacity of the virus. A prediction of this model is that if lower temperatures negatively affect virus replication more than RNAi, the extrinsic incubation period will increase with decreasing temperatures, a trend observed for most arbovirus-vector interactions [7], [8], [33]. In contrast, for virus vector combinations where RNAi is adversely affected more than viral replication, virus production will decrease with increasing temperatures (ie, increased modulation by RNAi). This scenario is consistent with observations previously reported for WEEV and RRV, in which mosquitoes were unable to modulate viral infections at lower temperatures [12], [13], [14].

Current models of arboviral disease transmission consider temperature as it relates to parameters such as mosquito longevity; time to complete development, and the extrinsic incubation period following exposure to a given arbovirus [5], [34], [35]. However, our data re-emphasize the importance of temperature on mosquito physiology in the time *prior to* virus exposure. Thus, current models may be improved by also taking into consideration the microclimates present in shaded or secluded breeding sites, such as those preferred by *Aedes* mosquitoes [4], [36]. For example, during the epidemic dengue transmission season in Buenos Aires (Jan-Mar), temperatures in shaded microenvironments were estimated to be ∼10°C cooler (22–25°C vs 30–37°C) than those in sunlit areas [37]. Similarly, during an outbreak of CHIKV in La Réunion, *Ae. albopictus* were found to prefer shaded breeding sites with average temperatures as low as 12.6°C [38]. During the same epidemic, a single amino acid substitution is known to have altered the infectivity of a CHIKV strain for *Ae. albopictus*, a non-traditional vector for this virus [39], [40]. Transient, temperature-induced increases in infectivity similar to those demonstrated here may increase opportunities for arboviruses to acquire adaptive mutations that permanently modify infectivity for a specific vector species. Urban epidemics resulting from the introduction of arboviral pathogens into densely populated areas are highly dependent on transmission by infected peridomestic vector species. Disconcertingly, we have shown that the infectivity of YFV and CHIKV for two important peridomestic vector species is improved when the aquatic stages of the insects' lifecycles occur at an environmentally relevant low temperature. Thus, our results, as well those published by others [9], [10], [11], suggest that temperature-dependent effects on vector competence may be applicable to many *Aedes* species and for alpha-, flavi-, and bunyaviruses.

Finally, we note that strategies relying on RNAi to generate transgenic mosquito strains with pathogen-resistant phenotypes have been in development for a considerable amount of time [41], [42]. Our data suggest that RNAi-based effecter genes may need to be re-evaluated for temperature-dependent effects on pathogen-resistance.

## Supporting Information

Figure S1 **CHIKV infection following rearing at 18°C or 28°C.** Infectivity of CHIKV for *Ae. aegypti* “sensor” strain following *per os* challenge (single replicate). The number of individuals examined is indicated above each bar.
(TIF)

## References

[1] Confalonieri U, Menne R, Akhtar KL, Ebi KL, Hauengue M, et al.. (2007) Human Heath. In: Parry ML, Canziani OF, Palutikof JP, van der Linden PJ, Hanson CE, editors. Climate Change 2007: Impacts, adaptation and vulnerability. Contribution of working group II to the fourth assessment report of the Intergovernmental Panel on Climate Change. Cambridge, U.K.: Cambridge University Press. pp. 391–431.

[2] Gubler DJ, Reiter P, Ebi KL, Yap W, Nasci R, et al (2001) Climate variability and change in the United States: potential impacts on vector- and rodent-borne diseases. Environ Health Perspect 109 Suppl 2: 223–233. 11359689 10.1289/ehp.109-1240669 PMC1240669

[3] Gould EA, Higgs S (2009) Impact of climate change and other factors on emerging arbovirus diseases. Trans R Soc Trop Med Hyg 103: 109–121. 18799177 10.1016/j.trstmh.2008.07.025 PMC2915563

[4] Reiter P (2008) Climate change and mosquito-borne disease: knowing the horse before hitching the cart. Rev Sci Tech 27: 383–398. 18819667

[5] Tabachnick WJ (2010) Challenges in predicting climate and environmental effects on vector-borne disease episystems in a changing world. J Exp Biol 213: 946–954. 20190119 10.1242/jeb.037564

[6] Thai KT, Anders KL (2011) The role of climate variability and change in the transmission dynamics and geographic distribution of dengue. Exp Biol Med (Maywood) 236: 944–954. 21737578 10.1258/ebm.2011.010402

[7] Davis NC (1932) The effect of various temperatures in modifying the extrinsic incubation period of the yellow fever virus in *Ae. aegypti* . Am J Hyg 16: 163–176.

[8] Hurlbut HS (1973) The effect of environmental temperature upon the transmission of St. Louis encephalitis virus by *Culex pipiens quinquefasciatus* . J Med Entomol 10: 1–12. 4697417 10.1093/jmedent/10.1.1

[9] Turell MJ, Lundstrom JO (1990) Effect of environmental temperature on the vector competence of *Aedes aegypti* and *Ae. taeniorhynchus* for Ockelbo virus. Am J Trop Med Hyg 43: 543–550. 2173434 10.4269/ajtmh.1990.43.543

[10] Turell MJ (1993) Effect of environmental temperature on the vector competence of *Aedes taeniorhynchus* for Rift Valley fever and Venezuelan equine encephalitis viruses. Am J Trop Med Hyg 49: 672–676. 8279634 10.4269/ajtmh.1993.49.672

[11] Westbrook CJ, Reiskind MH, Pesko KN, Greene KE, Lounibos LP (2009) Larval environmental temperature and the susceptibility of *Aedes albopictus* Skuse (Diptera: Culicidae) to chikungunya virus. Vector Borne Zoonotic Dis 10: 241–247. 10.1089/vbz.2009.0035 PMC2883477 19725768

[12] Kramer LD, Hardy JL, Presser SB (1983) Effect of temperature of extrinsic incubation on the vector competence of *Culex tarsalis* for western equine encephalomyelitis virus. Am J Trop Med Hyg 32: 1130–1139. 6625067 10.4269/ajtmh.1983.32.1130

[13] Kramer LD, Hardy JL, Presser SB (1998) Characterization of modulation of western equine encephalomyelitis virus by *Culex tarsalis* (Diptera: Culicidae) maintained at 32 degrees C following parenteral infection. J Med Entomol 35: 289–295. 9615548 10.1093/jmedent/35.3.289

[14] Kay BH, Jennings CD (2002) Enhancement or modulation of the vector competence of *Ochlerotatus vigilax* (Diptera: Culicidae) for Ross River virus by temperature. J Med Entomol 39: 99–105. 11931278 10.1603/0022-2585-39.1.99

[15] Hardy JL, Rosen L, Kramer LD, Presser SB, Shroyer DA, et al (1980) Effect of rearing temperature on transovarial transmission of St. Louis encephalitis virus in mosquitoes. Am J Trop Med Hyg 29: 963–968. 7435796 10.4269/ajtmh.1980.29.963

[16] Myles KM, Wiley MR, Morazzani EM, Adelman ZN (2008) Alphavirus-derived small RNAs modulate pathogenesis in disease vector mosquitoes. Proc Natl Acad Sci U S A 105: 19938–19943. 19047642 10.1073/pnas.0803408105 PMC2604946

[17] Campbell CL, Keene KM, Brackney DE, Olson KE, Blair CD, et al (2008) *Aedes aegypti* uses RNA interference in defense against Sindbis virus infection. BMC Microbiol 8: 47. 18366655 10.1186/1471-2180-8-47 PMC2278134

[18] Sanchez-Vargas I, Scott JC, Poole-Smith BK, Franz AW, Barbosa-Solomieu V, et al (2009) Dengue virus type 2 infections of *Aedes aegypti* are modulated by the mosquito's RNA interference pathway. PLoS Pathog 5: e1000299. 19214215 10.1371/journal.ppat.1000299 PMC2633610

[19] Morazzani EM, Wiley MR, Murreddu MG, Adelman ZN, Myles KM (2012) Production of virus-derived ping-pong-dependent piRNA-like small RNAs in the mosquito soma. PLoS Pathog 8: e1002470. 22241995 10.1371/journal.ppat.1002470 PMC3252369

[20] Vodovar N, Bronkhorst AW, van Cleef KW, Miesen P, Blanc H, et al (2012) Arbovirus-derived piRNAs exhibit a ping-pong signature in mosquito cells. PLoS One 7: e30861. 22292064 10.1371/journal.pone.0030861 PMC3265520

[21] Wang XH, Aliyari R, Li WX, Li HW, Kim K, et al (2006) RNA interference directs innate immunity against viruses in adult Drosophila. Science 312: 452–454. 16556799 10.1126/science.1125694 PMC1509097

[22] Mueller S, Gausson V, Vodovar N, Deddouche S, Troxler L, et al (2010) RNAi-mediated immunity provides strong protection against the negative-strand RNA vesicular stomatitis virus in Drosophila. Proc Natl Acad Sci U S A 107: 19390–19395. 20978209 10.1073/pnas.1014378107 PMC2984213

[23] Ding SW (2010) RNA-based antiviral immunity. Nat Rev Immunol 10: 632–644. 20706278 10.1038/nri2824

[24] Sabin LR, Hanna SL, Cherry S (2010) Innate antiviral immunity in Drosophila. Curr Opin Immunol 22: 4–9. 20137906 10.1016/j.coi.2010.01.007 PMC2831143

[25] Adelman ZN, Anderson MA, Morazzani EM, Myles KM (2008) A transgenic sensor strain for monitoring the RNAi pathway in the yellow fever mosquito, *Aedes aegypti* . Insect Biochem Mol Biol 38: 705–713. 18549956 10.1016/j.ibmb.2008.04.002 PMC2518454

[26] Myles KM, Kelly CLH, Ledermann JP, Powers AM (2006) Effects of an Opal Termination Codon Preceding the nsP4 Gene Sequence in the O'Nyong-Nyong Virus Genome on *Anopheles gambiae* Infectivity. J Virol 80: 4992–4997. 16641290 10.1128/JVI.80.10.4992-4997.2006 PMC1472075

[27] Anderson MA, Gross TL, Myles KM, Adelman ZN (2010) Validation of novel promoter sequences derived from two endogenous ubiquitin genes in transgenic *Aedes aegypti* . Insect Mol Biol 19: 441–449. 20456509 10.1111/j.1365-2583.2010.01005.x PMC3605713

[28] Plaskon NE, Adelman ZN, Myles KM (2009) Accurate strand-specific quantification of viral RNA. PLoS One 4: e7468. 19847293 10.1371/journal.pone.0007468 PMC2760750

[29] Langmead B, Trapnell C, Pop M, Salzberg SL (2009) Ultrafast and memory-efficient alignment of short DNA sequences to the human genome. Genome Biol 10: R25. 19261174 10.1186/gb-2009-10-3-r25 PMC2690996

[30] Chotkowski HL, Ciota AT, Jia Y, Puig-Basagoiti F, Kramer LD, et al (2008) West Nile virus infection of *Drosophila melanogaster* induces a protective RNAi response. Virology 377: 197–206. 18501400 10.1016/j.virol.2008.04.021 PMC2518314

[31] Szittya G, Silhavy D, Molnar A, Havelda Z, Lovas A, et al (2003) Low temperature inhibits RNA silencing-mediated defence by the control of siRNA generation. Embo J 22: 633–640. 12554663 10.1093/emboj/cdg74 PMC140757

[32] Chellappan P, Vanitharani R, Ogbe F, Fauquet CM (2005) Effect of temperature on geminivirus-induced RNA silencing in plants. Plant Physiol 138: 1828–1841. 16040661 10.1104/pp.105.066563 PMC1183375

[33] Watts DM, Burke DS, Harrison BA, Whitmire RE, Nisalak A (1987) Effect of temperature on the vector efficiency of *Aedes aegypti* for dengue 2 virus. Am J Trop Med Hyg 36: 143–152. 3812879 10.4269/ajtmh.1987.36.143

[34] Yang HM, Macoris ML, Galvani KC, Andrighetti MT, Wanderley DM (2009) Assessing the effects of temperature on dengue transmission. Epidemiol Infect 137: 1179–1187. 19192323 10.1017/S0950268809002052

[35] Descloux E, Mangeas M, Menkes CE, Lengaigne M, Leroy A, et al (2012) Climate-based models for understanding and forecasting dengue epidemics. PLoS Negl Trop Dis 6: e1470. 22348154 10.1371/journal.pntd.0001470 PMC3279338

[36] Barrera R, Amador M, Clark GG (2006) Ecological factors influencing *Aedes aegypti* (Diptera: Culicidae) productivity in artificial containers in Salinas, Puerto Rico. J Med Entomol 43: 484–492. 16739405 10.1603/0022-2585(2006)43[484:efiaad]2.0.co;2

[37] Vezzani D, Albicocco AP (2009) The effect of shade on the container index and pupal productivity of the mosquitoes *Aedes aegypti* and *Culex pipiens* breeding in artificial containers. Med Vet Entomol 23: 78–84. 19239617 10.1111/j.1365-2915.2008.00783.x

[38] Delatte H, Dehecq JS, Thiria J, Domerg C, Paupy C, et al (2008) Geographic distribution and developmental sites of *Aedes albopictus* (Diptera: Culicidae) during a chikungunya epidemic event. Vector Borne Zoonotic Dis 8: 25–34. 18171104 10.1089/vbz.2007.0649

[39] Tsetsarkin KA, Vanlandingham DL, McGee CE, Higgs S (2007) A single mutation in chikungunya virus affects vector specificity and epidemic potential. PLoS Pathog 3: e201. 18069894 10.1371/journal.ppat.0030201 PMC2134949

[40] Vazeille M, Moutailler S, Coudrier D, Rousseaux C, Khun H, et al (2007) Two chikungunya isolates from the outbreak of La Reunion (Indian Ocean) exhibit different patterns of infection in the mosquito, *Aedes albopictus* . PLoS One 2: e1168. 18000540 10.1371/journal.pone.0001168 PMC2064959

[41] Olson KE, Higgs S, Gaines PJ, Powers AM, Davis BS, et al (1996) Genetically engineered resistance to dengue-2 virus transmission in mosquitoes. Science 272: 884–886. 8629025 10.1126/science.272.5263.884

[42] Franz AW, Sanchez-Vargas I, Adelman ZN, Blair CD, Beaty BJ, et al (2006) Engineering RNA interference-based resistance to dengue virus type 2 in genetically modified *Aedes aegypti* . Proc Natl Acad Sci U S A 103: 4198–4203. 16537508 10.1073/pnas.0600479103 PMC1449670

