# Supplementary figures and images for: Cooler Temperatures Destabilize RNA Interference and Increase Susceptibility of Disease Vector Mosquitoes to Viral Infection
Source: PLoS Negl Trop Dis. 2013 May 30;7(5):e2239. doi: 10.1371/journal.pntd.0002239 (PMC3667787; doi:10.1371/journal.pntd.0002239)

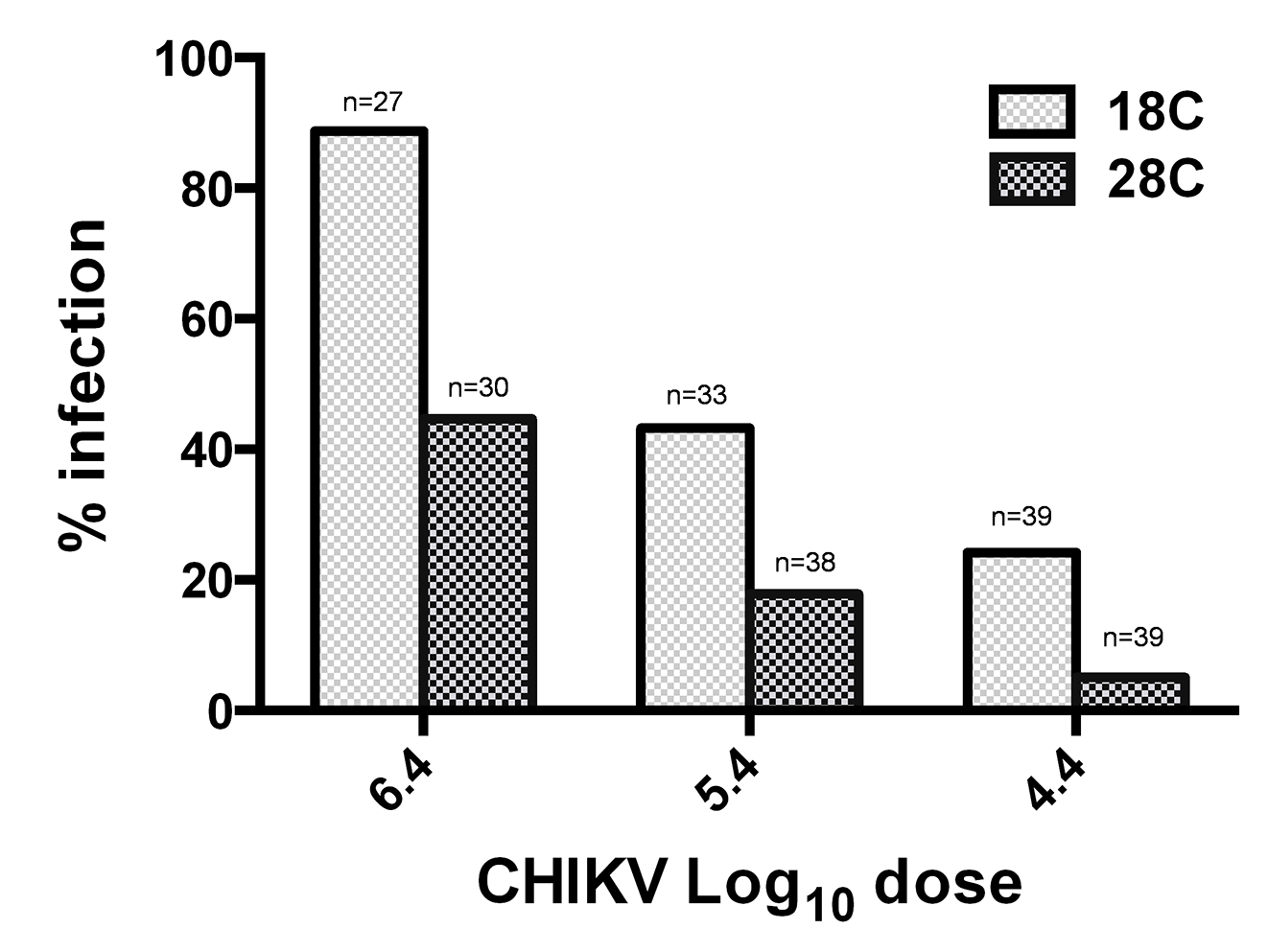

Supplement: Figure S1 — CHIKV infection following rearing at 18°C or 28°C. Infectivity of CHIKV for Ae. aegypti “sensor” strain following per os challenge (single replicate). The number of individuals examined is indicated above each bar. (TIF) [file pntd.0002239.s001.tif]
